# The teneurin C-terminal domain possesses nuclease activity and is apoptogenic

**DOI:** 10.1242/bio.031765

**Published:** 2018-03-15

**Authors:** Jacqueline Ferralli, Richard P. Tucker, Ruth Chiquet-Ehrismann

**Affiliations:** 1 Friedrich Miescher Institute for Biomedical Research, Novartis Research Foundation, Basel CH-4058, Switzerland; 2 Department of Cell Biology and Human Anatomy, University of California, Davis, California 95616-8643, United States of America; 3 Faculty of Science, University of Basel, Basel CH-4056, Switzerland

**Keywords:** Teneurin, Odz, Development, Apoptosis, Endonuclease, ABC complex

## Abstract

Teneurins are type 2 transmembrane proteins expressed by developing neurons during periods of synaptogenesis and apoptosis. Neurons expressing teneurin-1 synapse with other teneurin-1-expressing neurons, and neurons expressing teneurin-2 synapse with other teneurin-2-expressing neurons. Knockdowns and mutations of teneurins lead to abnormal neuronal connections, but the mechanisms underlying teneurin action remain unknown. Teneurins appear to have evolved via horizontal gene transfer from prokaryotic proteins involved in bacterial self-recognition. The bacterial teneurin-like proteins contain a cytotoxic C-terminal domain that is encapsulated in a tyrosine-aspartic acid repeat barrel. Teneurins are likely to be organized in the same way, but it is unclear if the C-terminal domains of teneurins have cytotoxic properties. Here we show that expression of teneurin C-terminal domains or the addition of purified teneurin C-terminal domains leads to an increase in apoptosis *in vitro*. The C-terminal domains of teneurins are most similar to bacterial nucleases, and purified C-terminal domains of teneurins linearize pcDNA3 and hydrolyze mitochondrial DNA. We hypothesize that yet to be identified stimuli lead to the release of the encapsulated teneurin C-terminal domain into the intersynaptic region, resulting in programmed cell death or the disruption of mitochondrial DNA and the subsequent pruning of inappropriate contacts.

## Abstract (precis)

**Summary:** Teneurins are transmembrane proteins found in the developing nervous system that are related to bacterial toxins. Teneurins also have cytotoxic properties that may help regulate apoptosis or pruning.

## INTRODUCTION

**Fig. 1.**
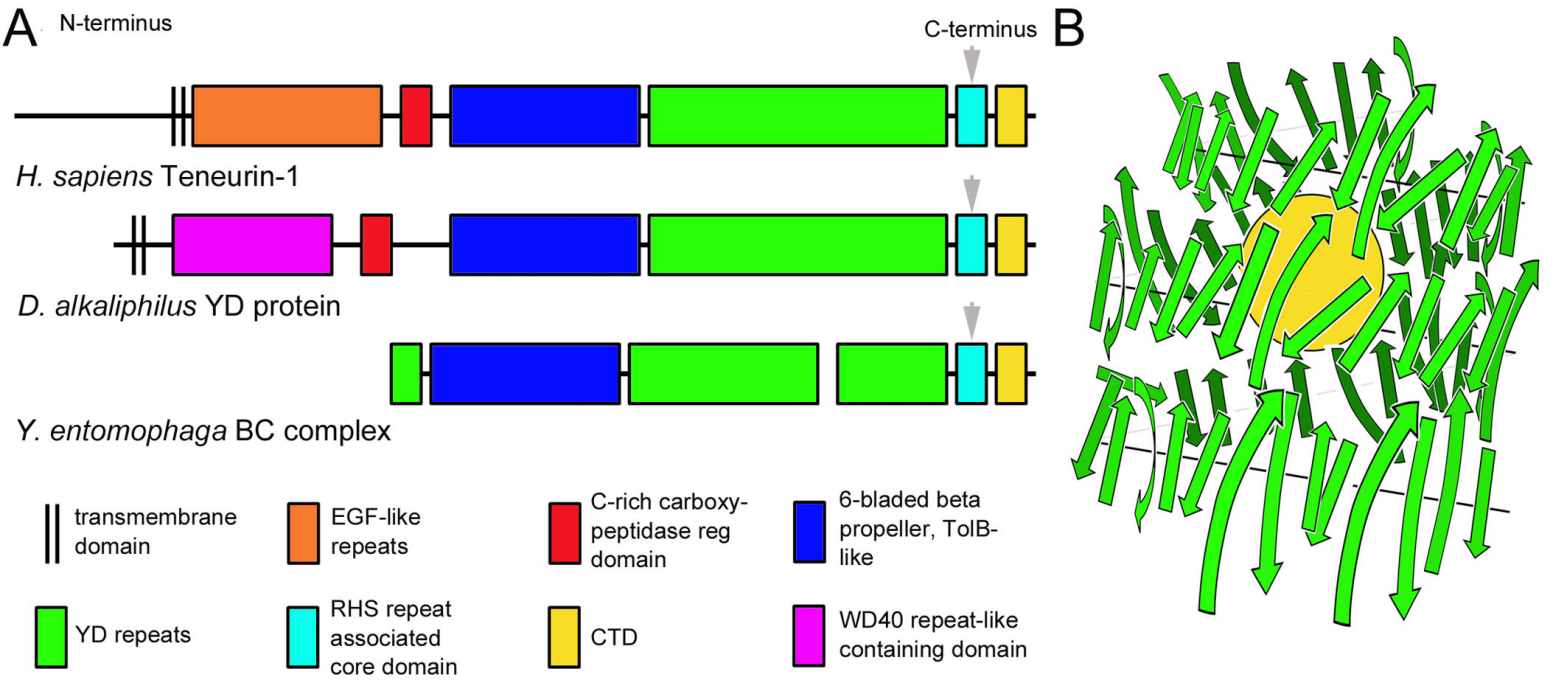
**Teneurins and related prokaryotic proteins.** (A) Teneurins are type 2 transmembrane proteins with the N-terminus inside the cell and the C-terminus outside the cell. The extracellular domain is composed of a series of EGF-like repeats (orange), a cysteine-rich domain (red), a six-bladed β-propeller (dark blue), and YD repeats (green). Near the C-terminus are an RHS repeat associated core-like domain (light blue) with a predicted furin cleavage site (arrow) and a C-terminal domain (CTD; yellow). Some prokaryotic YD proteins (e.g. *Desulfurivibrio alkaliphilus* YD protein) have a domain organization that is similar to that of teneurins, as do BC complex proteins (e.g. the *Yersinia entomophaga* BC complex). The latter are often expressed as two separate proteins that form a complex after they are secreted. Proteolytic cleavage sites (arrows) are found in the RHS repeat-associated core domains of the bacterial proteins, and the CTD of both the YD proteins and BC complex proteins are toxins. (B) Others (Busby et al., 2013) have shown that the YD repeats (green arrows) found in the BC proteins of *Y. entomophaga* make a barrel that envelops the toxic CTD (yellow sphere). The toxin is released when the barrel is disrupted. The green arrows representing the YD repeats indicate the N-terminal to C-terminal orientation of each repeat. The YD repeats, RHS repeat and CTD of teneurins are likely to be folded in the same way, encapsulating the CTD.

Teneurins are type 2 transmembrane proteins, first discovered in *Drosophila* (Baumgartner and Chiquet-Ehrismann, 1993; Levine et al., 1994), that are expressed in the nervous system of vertebrates during periods of synaptogenesis and programmed cell death (Tucker and Chiquet-Ehrismann, 2006; Young and Leamey, 2009; Leamey and Sawatari, 2014; Mosca, 2015). The extracellular region of teneurins has a domain organization that is highly conserved across bilateria, with eight epidermal growth factor (EGF)-like repeats that support dimerization (Oohashi et al., 1999), a cysteine-rich region with homology to the carboxypeptidase regulatory domain, a homophilic interacting six-bladed β-propeller (Tucker et al., 2012; Beckmann et al., 2013), a long stretch of tyrosine-aspartic acid (YD) repeats (Minet and Chiquet-Ehrismann, 2000), a rearrangement hot spot (RHS)-associated core protein-like domain with a predicted furin cleavage site (Tucker et al., 2012), and a C-terminal domain (CTD) with predicted homology to prokaryotic toxins (Zhang et al., 2012) (Fig. 1A). Most animals have multiple teneurin genes, with two identified in *Drosophila* (ten^a^ and ten^m^) and four in tetrapods (teneurins-1 through -4) (Tucker et al., 2012). In *Drosophila*, neurons expressing ten^a^ form synapses with other neurons expressing ten^a^, and neurons expressing ten^m^ synapse with other ten^m^-expressing neurons (Hong et al., 2012). In the chicken embryo, neurons of the thalamofugal visual system express teneurin-2, while neurons of the tectofugal visual system express teneurin-1 (Kenzelmann et al., 2008). Knockdown and misexpression studies in a variety of model systems, as well as genetic analysis of humans with sensory anomalies, illustrate the importance of normal teneurin expression for the formation of normal neuronal networks (Hong et al., 2012; Antinucci et al., 2013; Merlin et al., 2013; Young et al., 2013; Tran et al., 2015; Alkelai et al., 2016). The molecular mechanisms underlying how teneurins promote appropriate neuron-neuron interactions, however, remain unclear.

Phylogenetic analysis reveals that teneurins likely evolved through horizontal gene transfer from a bacterium into an ancestral choanoflagellate, with the eukaryotic proteins being formed through the fusion of a bacterial gene encoding a cysteine-rich domain, a six-bladed β-propeller, YD repeats, an RHS core-associated domain and a CTD with a eukaryotic gene encoding a type-2 transmembrane protein with EGF-like repeats (Tucker et al., 2012). Recent advances in our understanding of the structure and function of the teneurin-like prokaryotic proteins have generated significant insights into the structure and possible function of teneurins themselves. The prokaryotic proteins with YD repeats tend to be either type-2 transmembrane proteins that play a role in self-recognition between bacteria (Zhang et al., 2012; Koskiniemi et al., 2013) or part of the ABC complex of secreted toxins that contribute to eukaryotic pathogenicity (Zhang et al., 2012; Hachani et al., 2014). Some transmembrane prokaryotic YD proteins are remarkably similar to teneurins in their domain architecture, though other domains substitute for the EGF-like repeats seen in teneurins (Fig. 1A). It has been hypothesized that when identical YD proteins interact through their six-bladed β-propellers the C-terminal toxin domain is not released, but the toxin domain is released following heterotypic interactions. The toxins found in the CTD are diverse and include deaminases, nucleases and peptidases (Zhang et al., 2012). The secreted BC complex of the ABC toxin in the bacterium *Yersinia entomophaga* is homologous in organization to the extracellular region of metazoan teneurins from the six-bladed β-propeller distal to the CTD (Fig. 1A). Elegant structural studies revealed that the YD repeats of this toxin form a hollow barrel, with the β-propeller exposed at one end and the RHS repeat associated core domain and toxic CTD stuffed into the other end (Busby et al., 2013) (Fig. 1B). The RHS domain of the BC proteins is cleaved as the protein folds, leaving the toxin free to diffuse away from a disrupted YD barrel (Busby et al., 2013). Thus, the cytotoxic CTD is encapsulated, protecting the bacterium and allowing for yet-to-be determined structural changes to cause the timely release of the toxin, either into the extracellular space or after uptake of the YD barrel into a target eukaryotic cell.

Is the CTD of teneurins, like the CTD of teneurin-like YD proteins, cytotoxic? Here we report the effects of the CTD of teneurin-1 and teneurin-2 on cell survival and further characterize the nature of its toxicity. Our observations lead us to predict that teneurins may act in the developing brain in a manner similar to their prokaryotic precursors.

## RESULTS

### The teneurin C-terminal domain is cytotoxic

**Fig. 2.**
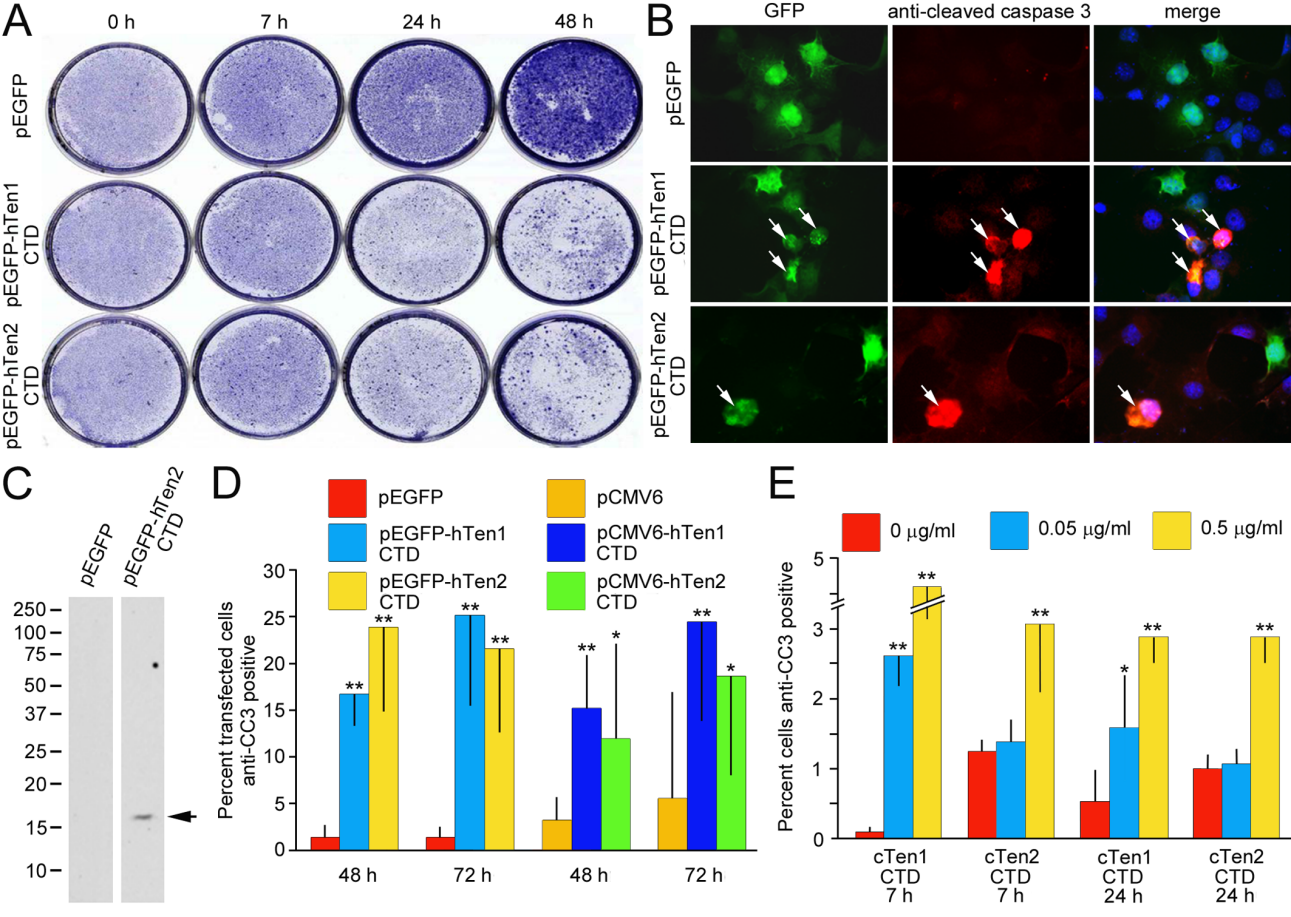
**The teneurin-1 and teneurin-2 C-terminal domains are apoptogenic.** (A) HEK 293 cells transfected with pEGFP alone or pEGFP with the CTD from human teneurin-1 (pEGFP-hTen1 CTD) or human teneurin-2 (pEGFP-hTen2 CTD) stained with Crystal Violet at different times following transfection. The control cultures become more densely stained over time, but not the cultures with cells expressing the CTDs. (B) Some of the cells transfected with the CTDs of teneurin-1 and teneurin-2 are immunostained with anti-cleaved caspase-3, a marker of apoptosis (arrows). (C) The anti-cleaved caspase-3 recognizes an appropriately sized band on an immunoblot of lysates from cells 7 h after being transfected with the teneurin-2 CTD (arrow). (D) A significantly higher percentage of HEK 293 cells expressing the EGFP-tagged CTDs are immunostained with anti-cleaved caspase-3 than controls at both 48 h and 72 h (*n*=3). Similarly, a higher percentage of COS-7 cells expressing myc-tagged CTDs are cleaved caspase-3-positive at these time points than cells transfected with pCMV6-A-puro alone (*n*=3). (E) The purified CTDs of both chicken teneurin-1 and chicken teneurin-2 cause a significant increase in the number of COS-7 cells that are positive for anti-cleaved caspase-3 when added to the culture medium (*n*=3). Error bars indicate mean±s.d. *P<0.05, **P<0.01, compared with controls (Student's *t*-test).

To determine if the teneurin CTD is cytotoxic, HEK 293 cells were transfected with a plasmid expressing EGFP-linked human teneurin C-terminal sequences. The expressed sequences correspond to most of the RHS repeat-associated core-like domain including the predicted furin cleavage site and the entire CTD. The expressed protein corresponds to the part of the teneurin that would be released from a disrupted YD barrel if the protein is processed at the predicted furin cleavage site (for details see Materials and Methods and Fig. S1). For clarity, the expressed proteins are referred to below as ‘CTD’. The cells were allowed to grow for different periods up to 48 h, then fixed and stained with Crystal Violet. The density of the staining of control and experimental cultures is similar for the first 7 h, but fewer cells appear to be present in the cultures expressing EGFP-linked CTDs at later time points (Fig. 2A). Next we determined if the apparent loss of cells was due to apoptosis by immunostaining the cultures with an antibody to the cleaved form of caspase-3 (Fig. 2B). At 7 h this antibody recognizes a band of the appropriate molecular mass on immunoblots of lysates of CTD expressing cells, but not control cells (Fig. 2C). Cell counts show a statistically significant increase in the number of anti-cleaved caspase-3-expressing cells in transfected cells after both 48 h and 72 h; similar observations are made in COS-7 cells transfected with an expression construct designed to generate teneurin CTD sequences fused with a myc tag instead of the EGFP tag (Fig. 2D). Note that controls used in these experiments were cells expressing the empty vectors and not cells expressing an unrelated protein of similar size to the CTDs.

Next we determined if purified chicken teneurin CTDs are cytotoxic when added to tissue culture medium. The CTD from teneurin-1 at final concentrations as low as 0.05 µg/ml (∼4 nM), cause a significant increase in the proportion of cells that are positive for anti-cleaved caspase-3 after just 7 h, but higher concentrations are needed to see significant effects with the CTD of teneurin-2 (Fig. 2E).

**Fig. 3.**
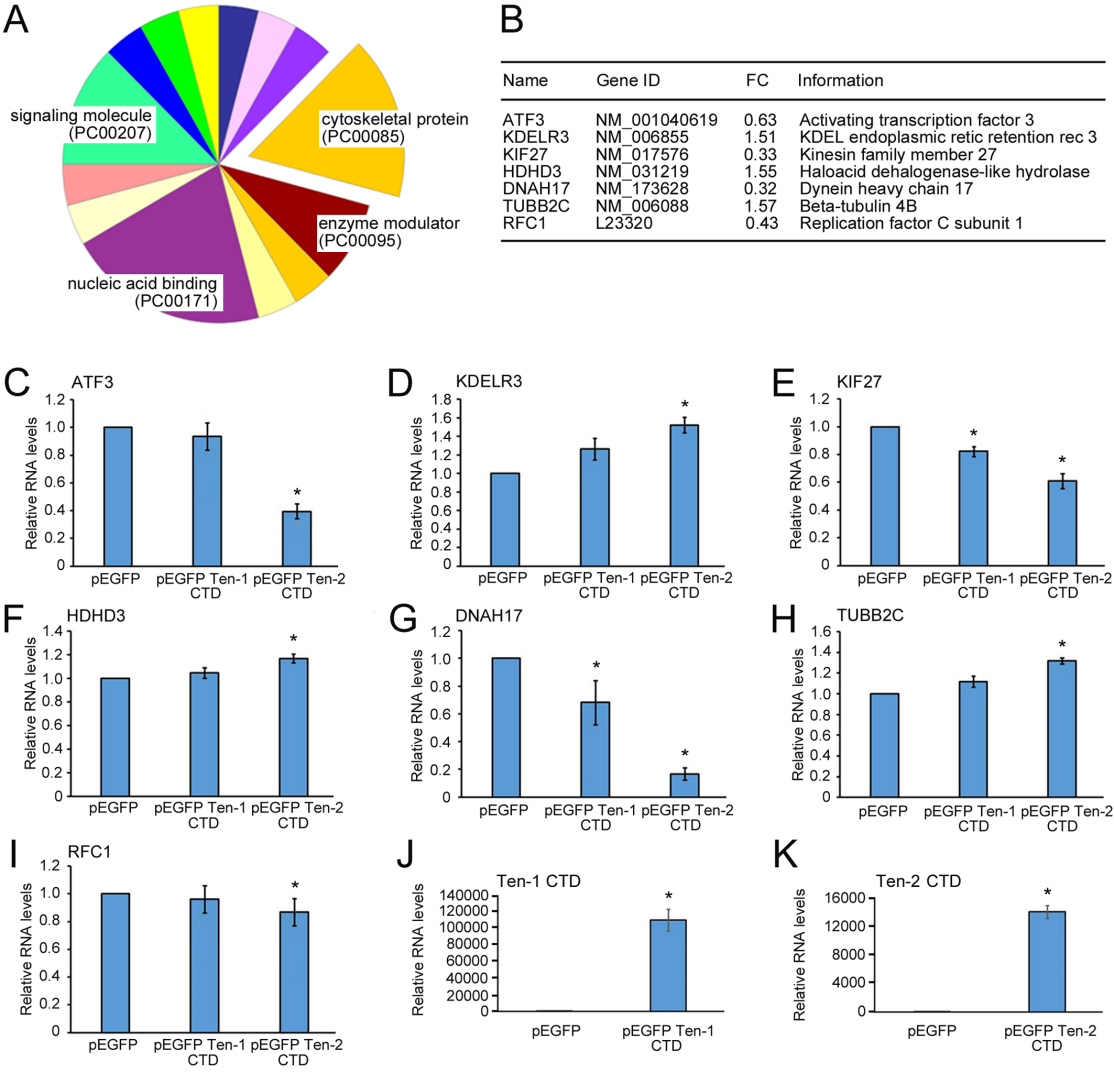
**Transcriptional profiling of HEK 293 cells expressing the C-terminal domain of teneurin-2 and confirmation by qRT-PCR.** (A) The genes that were down-regulated in HEK 293 cells expressing the CTD from teneurin-2 were analyzed using online tools from the Gene Ontology Consortium. Many of the genes were related to the cytoskeleton and vesicle transport, enzyme modulation, cell signaling and nucleic acid binding. (B) A table listing seven representative genes that were either up-regulated or down-regulated in the HEK 293 cells following expression of the C-terminal domain of teneurin-2 showing the fold change (FC), their gene identification number and their common name. A complete list of up- and down-regulated genes is included as supplemental information (Table S1). (C-I) The changes found by transcriptional profiling of the representative genes were confirmed with qRT-PCR. (J,K) qRT-PCR also confirmed the overexpression of the CTDs from teneurin-1 and teneurin-2 in the HEK 293 cells used in the analysis. *P<0.05.

cDNAs made from RNA isolated from sorted HEK 293 cells expressing EGFP-tagged CTDs from teneurins-2 and control cells were used for transcriptional profiling (Table S1). Transcripts that are significantly up- or down-regulated include a DNA repair enzyme (RFC1) and factors that others identified in neurons induced to undergo apoptosis (ATF3, HDHD3) (Kristiansen et al., 2011; Chen et al., 2014). These and other changes were confirmed with RT-qPCR (Fig. 3).

### Phylogenetic analysis of the teneurin C-terminal domain

**Fig. 4.**
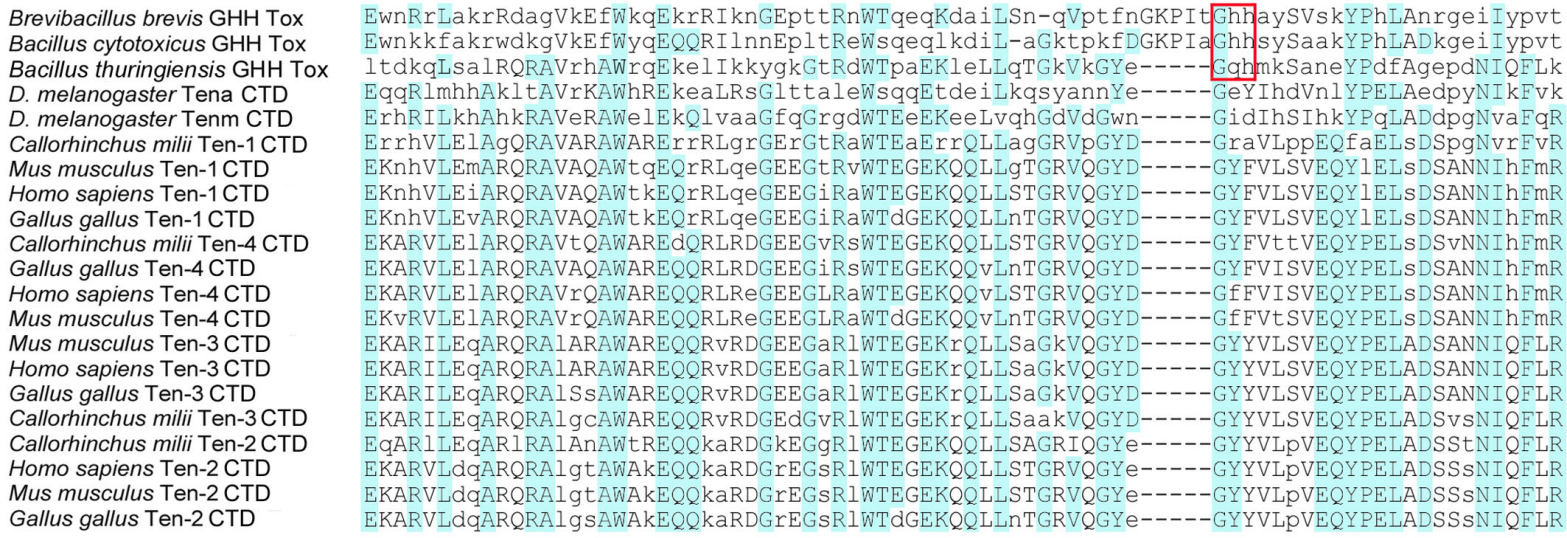
**The C-terminal domains of teneurins are most similar to the GHH toxin domains of bacterial proteins.** The GHH toxin domains from three bacterial (*Brevibacillus brevis*, *Bacillus cytotoxicus*, and *Bacillus thuringiensis*) proteins and the CTDs of representative teneurins aligned using Muscle 3.7, with residues with a BLOSUM62 score greater than 0.5 capitalized, and residues with a high (3.0) BLOSUM62 score highlighted in blue. Teneurin sequences used in the analysis come from *Homo sapiens*, *Mus musculus* (house mouse), *Gallus gallus* (chicken), *Callorhincus milli* (elephant shark) and the fruit fly *Drosophila melanogaster*, and correspond to the sequences found just C-terminal to the predicted furin cleavage site to five to 10 residues from the C-terminus of the protein. The GHH (or GQH) residues that give their name to the toxin domain in the bacterial proteins are boxed in red; these are not conserved in teneurins, but the adjacent GYE/D motif is found in the GHH toxin from *Bacillus thuringiensis* and all of the vertebrate teneurin CTDs.

Alignment of the sequences reveals the degree of identity and similarity between the GHH toxins of representative bacterial proteins and the teneurin CTDs (Fig. 4). For example, the amino acid sequence of the GHH toxin of a teneurin-like YD repeat protein from *Brevibacillus brevis* and the CTD of human teneurin-1 are 23.37% identical and 28.57% similar, and the amino acid sequence of the GHH toxin domain of a non-YD repeat protein with a GHH toxin domain from *Bacillus thuringiensis* and the CTD of human teneurin-2 is 37.66% identical and 48.05% similar. Among tetrapods the CTD amino acid sequence is strongly conserved. For example, the 77 amino acid-long portion of the CTDs from human and chicken teneurin-1 that align with the GHH toxins share all but three amino acids. Matrices showing the identity and similarity of these domains are found in the supplemental information (Fig. S2).

### The teneurin C-terminal domain has nuclease activity

**Fig. 5.**
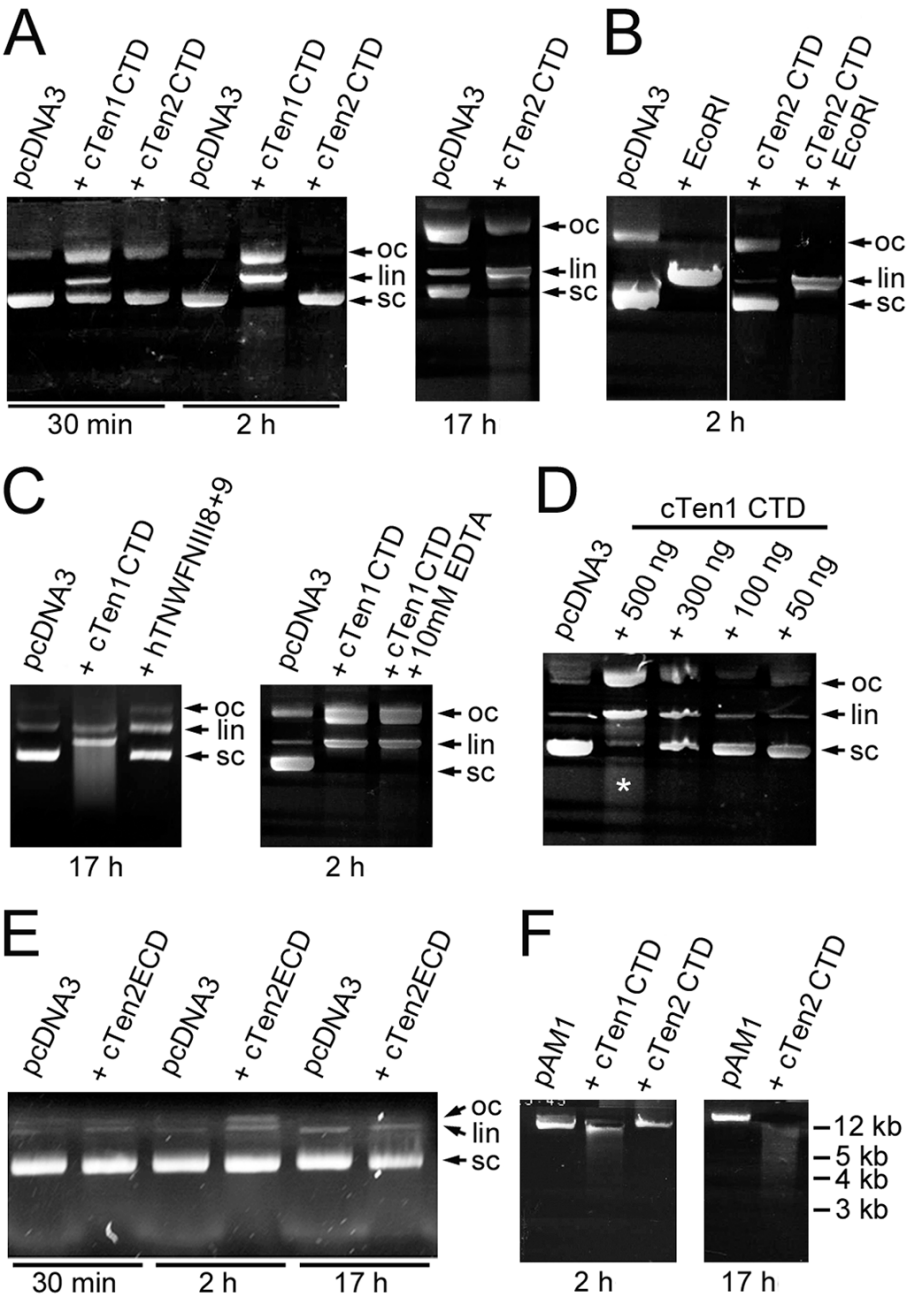
**The C-terminal domains** **of teneurin-1 and teneurin-2 have endonuclease activity.** (A) The CTD of teneurin-1 (cTen1 CTD) and teneurin-2 (cTen2 CTD) can linearize pcDNA3, with this effect first apparent after 30 min with the teneurin-1 CTD and after an overnight incubation with the teneurin-2 CTD. Open coil (oc), linearized (lin) and super coiled (sc) DNA are indicated with arrows. (B) The teneurin-2 CTD does not have exonuclease activity, at least following 2 h incubations with pcDNA3 previously linearized with EcoRI. (C) A recombinant protein (hTNWFNIII8+9) expressed and purified using the same conditions as the CTDs does not linearize pcDNA3 after an overnight incubation. Potentially contaminating Type I and Type II restriction endonucleases should not cleave pcDNA3 in the presence of 10 mM EDTA. However, 10 mM EDTA does not inhibit the endonuclease activity of the GHH toxin-like CTD from teneurin-1, which is a property expected from an endonuclease related to GHH toxins. (D) To determine the best conditions for the study of the nuclease activity of the teneurin-1 CTD, 200 ng of plasmid pcDNA3 was incubated with various concentrations of purified teneurin-1 CTD for 2 h. The decrease in super coiled DNA and the subsequent increase in linear and open circle DNA indicate endonuclease activity, while the smear (asterisk) indicates the presence of exonuclease activity. (E) The extracellular domain of chicken teneurin-2 (cTen2 ECD) was purified and 500 ng was incubated with 200 ng of plasmid for up to 17 h. When expressed in a form where the CTD is expected to be encapsulated in a YD repeat barrel, there is no endonuclease activity. (F) A plasmid containing the complete mitochondrial DNA from mouse (pAM1) is hydrolyzed by the CTD from teneurin-1 after 2 h, and by the CTD from teneurin-2 after an overnight incubation. With the exception of the experiment shown in D, which was done only once, all other reactions were reproduced at least three times.

Bacterial GHH toxins belong to the HNH/Endo VII family of endonucleases. Unlike the commonly encountered Type I and Type II restriction endonucleases that require Mg^++^ to catalyze the enzymatic activity, HNH endonucleases typically cleave DNA in the presence of either Ca^++^ or Mg^++^ (Pommer et al., 2001; Nagamalleswari et al., 2012). As the GHH (or similar) amino acid motif, which is critical for the endonuclease activity of the bacterial toxins, has evolved into GYY or GYF in most vertebrate teneurins (Fig. 4), others have predicted that the teneurin CTD does not have endonuclease activity (Zhang et al., 2012). We chose to test this by incubating the purified chicken C-terminal GHH toxin-like domains of teneurin-1 and teneurin-2 with the plasmid pcDNA3, a simple experiment based on the methods used by others (de Roodt et al., 2003), to demonstrate that certain components in snake venom have endonuclease activity. After 30 min the plasmid is cleaved by the purified teneurin-1 CTD, resulting in the appearance of linearized DNA together with open coil DNA (Fig. 5A). The GHH toxin-like CTD of teneurin-2 does not cleave the plasmid after 2 h, but does after an overnight incubation (Fig. 5A). If the plasmid is first linearized with EcoRI and then incubated with the teneurin-2 CTD, the CTD fails to further cleave the linear DNA (Fig. 5B). Thus, in short term experiments the GHH toxin-like CTD of teneurin-2 fails to demonstrate either endonuclease or exonuclease activity, but it does exhibit endonuclease activity after longer incubation times. Controls included incubating the plasmid with an irrelevant protein of similar size that was purified using the same methods as the teneurin CTDs and incubating the teneurin-1 CTD with the plasmid in the presence of 10 mM EDTA. The irrelevant protein fails to cut the plasmid, and 10 mM EDTA, which should chelate the Mg^++^ needed for potentially contaminating Type I and Type II restriction endonucleases, does not inhibit the endonuclease activity of the CTD (Fig. 5C). This is consistent with the proposed relationship of the teneurin CTD to Ca^++^-dependent, and Mg^++^-independent, HNH/Endo VII family endonucleases. The sequence in pcDNA3 that is cleaved by the teneurin-1 CTD was determined by cloning linearized plasmid into the EcoRV site of pBluescript and sequencing with a T3 primer. The plasmid sequence that is cleaved (TACGACTCACTA|TAGGGAGACCCA) does not correspond to the sequence of any published restriction endonuclease. When the teneurin-1 CTD is incubated with the plasmid for longer periods (Fig. 5C), or when higher concentrations of the teneurin-1 CTD are incubated with the plasmid (Fig. 5D), the CTD appears to have exonuclease activity as well. To see if the CTD can cleave plasmid if it is encapsulated in the YD barrel, the entire extracellular domain of chicken teneurin-2 was purified and incubated with plasmid for up to 17 h. No nuclease activity was observed (Fig. 5E).

### The teneurin C-terminal domain cleaves mitochondrial DNA *in vitro*

As mitochondria are needed to develop and maintain synapses (Li et al., 2004; D'Amelio et al., 2012; Hermes et al., 2016), and mitochondria would provide a convenient prokaryotic DNA-like target for toxins released near or into the developing synaptic cleft (Jiang et al., 2012), CTDs from teneurin-1 and teneurin-2 were incubated with a plasmid containing the entire murine mitochondrial DNA (pAM1). The CTD of teneurin-1 hydrolyzes the plasmid after 2 h, and the same domain from teneurin-2 hydrolyzes the mitochondrial DNA-containing plasmid after incubation overnight (Fig. 5F). Thus, both teneurin-1 and teneurin-2 have C-terminal GHH toxin-like domains with the potential to degrade mitochondrial DNA and initiate either failure of synaptogenesis or apoptosis.

## DISCUSSION

The motif RXRR is found in all vertebrate teneurin sequences examined to date just C-terminal to the RHS domain. This motif is predicted in silico to be a cleaved by furin or a furin-like protease (Tucker et al., 2012), but there is meager experimental evidence supporting this prediction. Antibodies raised against the CTD of *Caenorhabditis elegans* ten-1 immunostained cell membranes in nematode embryos, but the antibodies did not label blots of embryo homogenates (Drabikowski et al., 2005). A polyclonal antibody raised against the extracellular domain of mouse teneurin-1, which may have included antibodies to the CTD, recognized both high molecular weight bands and a band running near the bottom of the gel on immunoblots of mouse brain homogenates (Oohashi et al., 1999). While the smaller band may represent processed CTD, future experiments need to address this issue before broader speculation about the role of the CTD in neuronal development can be made.

Many of the residues conserved between the bacterial toxin domains and the teneurin CTDs are charged (Fig. 4). Accordingly, others (Busby et al., 2013) found that the inner surfaces of the beta-strands that make up the encapsulating barrel (Fig. 1B) are hydrophobic. These authors speculated that since the inner surface of the YD-repeats of vertebrate teneurins are similarly hydrophobic, that the charged teneurin CTD is likely to be encapsulated in the barrel, just like the toxin is encapsulated in the barrel of homologous prokaryotic proteins.

While teneurins are homologous in organization to transmembrane bacterial YD proteins, the extracellular domain of teneurins from the β-propeller domain to the CTD is also remarkably similar to some BC proteins of the ABC toxin complex (e.g. the BC proteins of *Y. entomophaga* illustrated in Fig. 1A). BC toxin complexes interact with an A protein that can insert into a target cell membrane, thus facilitating the uptake of the toxin. Interestingly, at least one teneurin, teneurin-2, has been shown to bind to the synaptic protein latrophilin-1 (Silva et al., 2011). Latrophilins are G-protein-coupled receptors that were named for their affinity for black widow spider-derived α-latrotoxin (Ushkaryov et al., 2008). Future studies should be directed toward clarifying the roles of teneurin-latrophilin interactions during development, including the possibility that latrophilins could play a role similar to that of the A proteins found in the ABC toxin complex.

The GHH (or related) motif is necessary for the toxic endonuclease activity of the extant bacterial YD proteins that are most similar to teneurins, but teneurins lack this motif as well as some neighboring sequences (Fig. 4). This led others to conclude that the CTD of teneurins was unlikely to cleave DNA (Zhang et al., 2012). Perhaps the active nuclease site in teneurins is the GYD/E motif, which is found in the teneurins of all vertebrates examined here (and nearly aligns with the GHH motif of the bacterial toxins) as well as in the secreted GHH endonuclease from *Bacillus thuringiensis*, which is 37.66% identical to the CTD of human teneurin-2. Future studies could be directed at mutating this site to see if it is in fact required for the observed nuclease activity of the teneurin CTD. It should be noted, however, that this motif is not seen in the *Drosophila melanogaster* teneurins ten^a^ and ten^m^.

Others have found that the C-terminal 38-41 amino acids of human teneurins have sequence identity (up to 12%) with certain corticotropin-releasing factor peptides (Lovejoy et al., 2009), and that peptides corresponding to the C-terminal 38-41 amino acids of teneurins can modulate anxiety-related behavior and dendritic spine density in the hippocampus of rats following introduction of the peptide into the right lateral ventricle (Tan et al., 2011). These teneurin C-terminal associated proteins (TCAPs) have very different properties from the CTDs described here. For example, instead of inducing apoptosis when added to the medium of N38 cells, TCAPs protect the cells from necrosis by alkaline pH (Trubiani et al., 2007). In full-length teneurins, the TCAP sequence is C-terminal to, and does not overlap with, the region that alignment with YD proteins indicates is the likely site of DNA binding and nuclease activity within the CTD. Thus, there is no reason to expect that TCAPs have either nuclease activity or are cytotoxic. More importantly, TCAPs probably do not act as a functional domain of full length teneurins, as they can be transcribed independently (Chand et al., 2013).

Bacteria evolved toxins encapsulated in a YD barrel with a six-bladed β-propeller, as part of the struggle for limited resources between different strains and species. Following the acquisition of a YD repeat-containing protein by an ancestral choanoflagellate, the early metazoan genome was primed for conducting a similar sort of chemical warfare between neurons in the central nervous system. We believe that the results presented here demonstrate evidence of a heretofore unknown mechanism for regulating neuronal development.

## MATERIALS AND METHODS

### Phylogenetic analysis

Teneurin CTDs are identified as GHH toxin-like domains by Pfam (http://pfam.xfam.org). For example, in the case of human teneurin-1 (NP_001156750) the CTD corresponds to the 78 amino acids found between amino acids 2648 through 2725. The CTDs of human teneurin-1 and teneurin-2 (NP_001073897) were used in an NCBI tBlastn search of prokaryotic sequences to identify the most similar domains in bacteria. The most similar YD repeat proteins were GHH toxin containing proteins from *Brevibacillus brevis* (BAH45440) and *Bacillus cytotoxicus* (CP000764; 2072317-2072078). The most similar sequence overall was a secreted GHH toxin without YD repeats from *Bacillus thuringiensis* (AJH07449). The GHH toxin domains from these bacterial sequences were used in subsequent analyses. The CTDs of teneurin-1 through -4 sequences from human (*Homo sapiens*; XP_011529539; NP_001073897; NP_001073946; XP_011543235), mouse (*Mus musculus*; NM_011855; NM_011856; NP_035987; BAA77399), chicken (*Gallus gallus*; NP_990193; NP_989428; NP_001185466; XP_015136353), the elephant shark (*Callorhinchus milii*; XP_007893009; XP_007900206; XP_007894102; XP_0079009700) as well as ten^a^ and ten^m^ from the fruit fly (*Drosophila melanogaster*; NP_511137; CAA51678) were aligned using Muscle 3.7 at Phylogeny.fr. Percent amino acid identity and similarity was determined using the SIAS program (http://imed.med.ucm.es/Tools/sias.html) of the Immunomedicine Group of the Universidad Complutense Madrid using the default amino acid grouping, the Blosum62 substitution matrix, and the cost for creating the gap P_o_ set at 10 and the cost for extending the gap P_e_ set at 0.5. FASTA files used for the analyses are found in Table S2.

### C-terminal domain expression vectors, transfection and cell culture

All cloning was done using traditional methods and the following kits and reagents: KOD Hot Start DNA polymerase (Novagen, Darmstadt, Germany, 71086-3), PCR product purification kit (Qiagen, 28106), gel extraction kit (Qiagen, 28706), Rapid DNA Ligation kit (Roche, 11635379001), and High Capacity cDNA Reverse Transcription kit (Applied Biosystems, Foster City, USA, 4322171). Teneurin-1 and teneurin-2 CTDs (the 123 C-terminal amino acids of teneurin-1, and the 125 C-terminal amino acids of teneurin-2; Fig. S1) were cloned from human cerebellum cDNA (AMS Biotechnology, Abingdon, UK, SRR306844) with the following primers: Teneurin-1 CTD1 (forward: ATGTCACTGTGTCCCAGATGACTTC, reverse: AGGCAGTCTTTGGTGACGCAAAGGC); Teneurin-1 CTD2 (forward: ATGCGATCGCATGGAACAAAAACTATTTCTGAAGAAGATCTGTTTGCAGATATTCAGCTCCAG, reverse: ATGCGGCCGCTTACCTCCTGCCTATTTCGCTCTG); Teneurin-2 CTD1 (forward: ACCGTGTCCCAGCCCACGCTGCTGG, reverse: CTCCTGCTGAGCCATTCAGACAAGG); Teneurin-2 CTD2 (forward: ATGCGATCGCATGGAACAAAAACTTATTTCTGAAGAAGATCTGTTCACGAACATTGAGTTCCAG, reverse: ATGCGGCCGCTTACCTCTTTCCCATCTCATTCTG). Purified PCR products were digested with AsiSI/NotI and ligated into pCMV6-A-puro (Origene, Rockville, USA, PS100025). All expression plasmids described here and elsewhere were verified by sequencing. COS-7 cells (ATCC, CRL-1651) were transfected with jetPEI transfection reagent (Polyplus, Illkirch, France, 101-10N) using the manufacturer's protocols. Constructs with the same domains were prepared using pEGFPC1-Myc (Clontech, Saint-Germain-en-Laye, France) with the following primers: human teneurin-1 CTD (forward XhoI: ATCTCGAGAACAAAAACTTATTTCTGAAGAAGATCTGTTTGCAGATATTCAGCTCCAG, reverse BamHI: ATGGATCCTTACCTCCTGCCTATTTCGCTCTG); human teneurin-2 CTD (forward XhoI: ATCTCGAGAACAAAAACTTATTTCTGAAGAAGATCTGTTCACGAACATTGAGTTCCAG, reverse BamHI: ATGGATCCTTACCTCTTTCCCATCTCATTCTG). Purified products and vectors were digested with XhoI and BamHI prior to ligation. Expression vectors were transfected into HEK 293 cells (ATCC, CRL-1573, authenticated and passaged fewer than 10 times) as described above or below. COS-7 (ATCC, CRL-1651, authenticated and passaged fewer than 10 times) and HEK 293 cells were cultured in Dulbecco's Minimum Essential Medium (DMEM) containing 10% fetal bovine serum (FBS), 100 mg/ml penicillin and 100 mg/ml streptomycin.

### Crystal Violet staining

On day 1, 2.5×10^5^ HEK 293 cells were plated onto 3.5 cm tissue culture dishes in DMEM and 10% FBS. On day 2, the cells were transfected with 2 µg of the empty pEGFP vector or pEGFP vectors with inserts encoding the human teneurin-1 or -2 CTDs (see above) using jetPEI transfection reagent. Immediately following transfection one dish from each experimental group was fixed in 4% paraformaldehyde in phosphate buffered saline (PBS) for 30 min, then washed three times in PBS, then stained with 0.1% Crystal Violet for 1 h, gently rinsed in distilled water and allowed to air dry. These dishes represented time point ‘0 h’. Cells in the other dishes were cultured for 7 h, 24 h or 48 h and then processed in the same manner.

### Transcriptional profiling and quantitative RT-PCR

Next, 1.2×10^6^ HEK 293 cells were seeded in 10 cm tissue culture dishes in DMEM supplemented with 10% FBS. Cells were transfected with 12 µg of expression vector with jetPEI. After 16 h the cells were trypsinized and centrifuged at 1100 rpm for 3 min. The medium was removed and the cells were resuspended in 500 µl of PBS with 2% FBS, filtered (CellTrics; Partec, Goerlitz, Germany, 04-004-2327) and the green fluorescent cells were sorted. Then, 10×10^4^ sorted cells were collected and RNA was isolated. RNA profiling was conducted and the data analyzed as described previously (Asparuhova et al., 2015). Quantitative RT-PCR was used to confirm selected profiles using Platinum SYBR Green qPCR SuperMix-UDG (Thermo Fisher Scientific, 11733038) and methods described previously (Asparuhova et al., 2015) using the following primers: *KIF27* (forward: GTAATTAAGCGGGACCAGCA, reverse: AGGTCCTCAGCTTTCGGTTT); *RAB7A* (forward: GTGTTGCTGAAGGTTATCATCCT, reverse: GCTCCTATTGTGGCTTTGTACTG); *TUBB2C* (forward: GGACAACTTCGTTTTCGGTCA, reverse: CCTTTCTCACAACATCCAGCAC); *KDELR3* (forward: TCCCAGTCATTGGCCTTTCC, reverse: CCAGTTAGCCAGGTAGAGTGC); *HDHD3* (forward: ATACGACTGCTGACGTGGGAT, reverse: TCAGGCCGTAGTTGGGGAA); *AGAP5* (forward: ACATGCACCACATTCGTGA, reverse: GGATTGGCAGAAAGGCTAAA); *DNAH17* (forward: GTGGAGCAAGCTGATAGGCG, reverse: AAGTAAACCCCTTTGGACTTGAG); *RCF1* (forward: AAGGCTAGGAATTTGGCTGA, reverse: TAGGAGTTTGTTGGCACAGC); *hATF3* (forward: CGCTGGAATCAGTCACTG, reverse: GCTTCTCCGACTCTTTCT); human teneurin-1 CTD (forward: AGCCCTGTGCTTCAACATC, reverse: GCTGTCTGGCAATCTCCAA); human teneurin-2 CTD (forward: CGTGGAGCAATACCCAGAG, reverse: CCTCTTTCCCATCTCATTCTG). All quantitative RT-PCR samples were run in duplicate. A *P*-value ≤0.05 was used to determine statistical significance using a two-tailed Student's *t*-test.

### C-terminal domain expression and protein purification

For protein expression and purification, the CTDs of chicken teneurin-1 and teneurin-2 were cloned using previously described eukaryotic expression vectors (Beckmann et al., 2013) as template and the following primers: chicken teneurin-1 CTD (forward BamHI: ATGGATCCTTCGCTGACATTCAGCTGCAGCATG, reverse HindIII: ATAAGCTTTTAACGGCGGCCGATTTCACTCTGACGCATAAAGTG); chicken teneurin-2 CTD (forward BamHI: ATGGATCCTTCACAAACATCGAGTTTCAGTATTC, reverse HindIII: ATAAGCTTTTACCTCTTTCCCATTTCATTCTGTC). Purified products were digested with BamHI/HindIII and cloned into pQE-30 (Qiagen). Proteins were expressed and purified as described previously (Degen et al., 2007). The protein appeared as a single band following SDS-PAGE and staining with Coomassie Blue (Fig. S3). The cloning and purification of the chicken teneurin-2 extracellular domain was described previously (Beckmann et al., 2013).

### Immunoblotting

Transfected HEK 293 cells were lysed in 4× sample loading buffer and separated by SDS-gel electrophoresis on a 12.5% polyacrylamide gel and electroblotted to polyvinylidene difluoride membranes (Thermo Fisher Scientific, LC2002). Similar loading and transfer of proteins were confirmed by staining the membranes with Amido Black. After a 1 h blocking step in 5% milk powder in Tris-buffered saline with 0.05% Tween-20, membranes were incubated overnight with a rabbit polyclonal antibody to cleaved caspase-3 (Asp175) diluted 1:1000 (Cell Signaling Technology, 9661S). After incubation for 1 h with anti-rabbit IgG coupled to horseradish peroxidase (MP Biomedicals, Burlingame, USA, 0855689), blots were developed using SuperSignal (Thermo Fisher Scientific, 34080) followed by exposure to Kodak BioMax MR Films (Sigma-Aldrich, Z350397).

### Immunocytochemistry and analysis of C-terminal domain induced apoptosis

COS-7 or HEK 293 cells were seeded at 8×10^4^ cells per 3.5 cm dish in DMEM with 10% FBS and transfected as described above. Alternatively, COS-7 cells were seeded and purified CTDs from teneurin-1 or teneurin-2 were added to the medium at various concentrations. Cell cultures were fixed at various time points following transfection or the addition of purified proteins with 4% paraformaldehyde in PBS for 30 min, washed twice in PBS, permeabilized for 5 min with 0.1% Triton in PBS, and rinsed again twice in PBS. Diluted anti-cleaved caspase-3 (see above) was added for 1.5 h at room temperature and the samples were then rinsed four times with PBS. For cultures expressing myc-tagged proteins, cultures were also incubated with mouse anti-myc for 1.5 h and then rinsed. Samples were then incubated in goat anti-mouse Alexa Fluor 488 or goat anti-rabbit Alexa Fluor 568 secondary antibodies (Molecular Probes) for 1 h. Studies with secondary antibodies alone were done in parallel to control for background. After final rinses and staining with Hoechst 33258 the cultures were mounted with ProLong Gold antifade reagent (Molecular Probes, 736934) and examined and photographed with an Axioskop photomicroscope (Carl Zeiss, Oberkochen, Germany) fitted with an ORCA-ER digital camera (Hamamatsu, Hamamatsu City, Japan). To analyze apoptosis in the transfected cultures, 200 cells from each experimental condition were identified with the nuclear stain and then scored as either untransfected and positive or negative for anti-cleaved caspase-3, or transfected (EGFP or anti-myc positive) and positive or negative for anti-cleaved caspase-3. To analyze apoptosis in the cultures with the purified proteins added to the medium, 200 cells were identified with the nuclear stain and scored as positive or negative for the anti-cleaved caspase-3. Results from three independent experiments were analyzed using a Student's *t*-test (one-tailed, unpaired, equal variance).

### Endonuclease activity

We incubated 200 ng of pcDNA3 (Thermo Fisher Scientific) for 2 h with various concentrations of the teneurin-1 CTD in the presence of 1× EcoRI buffer (New England BioLabs, Ipswich, USA, B0101S) to determine the optimal conditions for further studies (Fig. 5D). Further reactions included the pcDNA3 plasmid mixed with 500 ng of the CTDs of chicken teneurins-1 and -2 at 37°C for different periods of time, predigestion of the plasmid with EcoRI (stopping the reaction by heating to 65°C for 20 min prior to the addition of the purified teneurin domains), and including 10 mM EDTA in the reaction mix. For an additional control, pcDNA3 was incubated with a similarly sized protein corresponding to the 8th and 9th fibronectin type III domains of human tenascin-W, which was expressed and purified using the same conditions as the CTDs (Degen et al., 2007), and with the entire extracellular domain of chicken teneurin-2 (Beckmann et al., 2013). The sequence within pcDNA3 that was cut by the toxin domain of teneurin-1 was determined by gel purification of the linearized plasmid followed by blunt cloning into the EcoRV site of pBluescript (Stratagene, San Diego, USA), followed by sequencing using a T3 primer. The plasmid containing the complete DNA of murine mitochondria, pAM1 (Martens and Clayton, 1979), was used for some reactions.

## Supplementary Material

Supplementary information
