# Supplementary material for: The teneurin C-terminal domain possesses nuclease activity and is apoptogenic
Source: Biol Open. 2018 Mar 15;7(3):bio031765. doi: 10.1242/bio.031765 (PMC5898268; doi:10.1242/bio.031765)
Supplement: Supplementary information [file biolopen-7-031765-s1.pdf]

Fig. S1. The domain organization of teneurin-1 and the sequences that were expressed or purified in this study. The amino acid sequence of human teneurin-1 with the various domains indicated. The expressed protein is underlined, the furin cleavage site is indicated in red, and the GHH toxin domain is in italics. The amino acid sequences of the two human and two chicken CTDs are also shown, with the furin cleavage sites in red and the region corresponding to the GHH toxin domain in italics.

Intracellular domain

MEQTDCKPYQPLPKVKHEMDLAYTSSSDESEDGRKPRQSYNSRETLHEYNQELRM
NYNSQSRKRKEVEKSTQEMEFCETSHTLCSGYQTDMHSVSRHGYQLEMGSDVDTE
TEGAASPDHALRMWIRGMKSEHSSCLSSRANSALSLTDTDHERKSDGENGFKFSP
VCCDMEAQAGSTQDVQSSPHNQFTFRPLPPPPPPPHACTCARKPPPAADSLQRRS
MTTRSQPSPAAPAPPTSTQDSVHLHNSWVLNSNIPLETRHFLFKHGSGSSAIFSA
ASQNYPLTSNTVYSPPPRPLPRSTFSRPAFTFNKPYRCCNWK

Transmembrane domain

CTALSATAITVTLALLLAYVIAV

Beginning of extracellular domain

HLFGLTWQLQPVEGELYANGVSKGNRGTESMDTTYSPIGGKVSDKSEKKVFQKGR
AIDTGEVDIGAQVMQTIPPGLFWRFQITIHHPIYLKFNISLAKDSLLGIYGRRNI
PPTHTQFDFVKLMDGKQLVKQDSKGSDDTQHSPRNLILTSLQETGFIEYMDQGPW
YLAFYNDGKKMEQVFVLTTAIEIM

EGF-like domains

DDCSTNCNGNGECISGHCHCFPGFLGPDCARDSCPVLCGGNGEYEKGHCVCRHGW
KGPECDVPEEQCIDPTCFGHGTCIMGVCICVPGYKGEICEEEDCLDPMCSNHGIC
VKGECHCSTGWGGVNCETPLPVCQEQCSGHGTFLLDAGVCSCDPKWTGSDCSTEL
CTMECGSHGVCSRGICQCEEGWVGPTCEERSCHSHCTEHGQCKDGKCECSPGWEG
DHCTIAHYLDAVRDGCPGLCFGNGRCTLDQNGWHCVCQVGWSGTGCNV

C-rich domain

VMEMLCGDNLDNDGDGLTDCVDPDCCQQSNCYISPLCQ

GSPDPLDLIQQSQTLFSQHTSRLFYDRIKFLIGKDSTHVIPPEVSFDSRRACVIR
GQVVAIDGTPLVGVNVSFLHHSDYGFTISRQDGSFDLVAIGGISVILIFDRSPFL
PEKRTLWLPWNQFIVVEKVTMQRVVSDPPSCDISNFISPNPIVLPSPLTSFGGSC
PERGTIVPELQVVQEEIPIPSSFVRLSYLSSRTPGYKTLLRILLTHSTIPVGMIK

Biology Open • Supplementary information

VHLTVAVEGRLTQKWFPAAINLVYTFAWNKTDIYGQKVWGLAEALVSVGYEYETC
PDFILWEQRTVVLQGFEMDASNLGGWSLNKHHILNPQSGIIHKGNGENMFISQQP
PV

6-bladed beta propeller
ISTIMGNGHQRSVACTNCNGPAHNNKLFAPVALASGPDGSVYVGDFNFVRRIFPS
GNSVSILELSTSPAHKYYLAMDPVSESLYLSDTNTRKVYKLKSLVETKDLSKNFE
VVAGTGDQCLPFDQSHCGDGGRASEASLNSPRGITVDRHGFIYFVDGTMIRKIDE
NAVITTVIGSNGLTSTQPLSCDSGMDITQVRLEWPTDLAVNPMDNSLYVLDNNIV
LQISENRRVRIIAGRPIHCQ

YD-repeats and first half of the RHS-domain
VPGIDHFLVSKVAIHSTLESARAISVSHSGLLFIAETDERKVNRIQQVTTNGEIY
IIAGAPTDCDCKIDPNCDCFSGDGGYAKDAKMKAPSSLAVSPDGTLYVADLGNVR
IRTISRNQAHLNDMNIYEIASPADQELYQFTVNGTHLHTLNLITRDYVYNFTYNS
EGDLGAITSSNGNSVHIRRDAGGMPLWLVVPGGQVYWLTISSNGVLKRVSAQGYN
LALMTYPGNTGLLATKSNENGWTTVYEYDPEGHLTNATFPTGEVSSFHSDLEKLT
KVELDTSNRENVLMSTNLTATSTIYILKQENTQSTYRVNPDGSLRVTFASGMEIG
LSSEPHILAGAVNPTLGKCNISLPGEHNANLIEWRQRKEQNKGNVSAFERRLRAH
NRNLLSIDFDHITRTGKIYDDHRKFTLRILYDQTGRPILWSPVSRYNEVNITYSP
SGLVTFIQRGTWNEKMEYDQSGKIISRTWADGKIWSYTYLEKSVMLLLHSQRRYI
FEYDQPDCLLSVTMPSMVRHSLQTMLSVGYYRNIYTPPDSSTSFIQDYSRDGRLL
QTLHLGTGRRVLYKYTKQARLSEVLYDTTQVTLTYEESSGVIKTIHLMHDGFICT
IRYRQTGPLIGRQIFRFSEEGLVNARFDYSYNNFRVTSMQAVINETPLPIDLYRY
VDVSGRTEQFGKFSVINYDLNQVITTTVMKHTKIFSANGQVIEVQYEILKAIAYW
MTIQYDNVGRMVICDIRVGVDANITRYFYEYDADGQLQTVSVNDKTQWRYSYDLN
GNINLLSHGKSARLTPLRYDLRDRITRLGEIQYKMDEDGFLRQRGNDIFEYNSNG
LLQKAYNKASGWTVQYYYDGLGRRVASKSSLGQHLQFFYADLTNPIRVTHLYNHT
SSEITSLYYDLQGHLIAMELSSGEEYYVACDNTGTPLAVFSSRGQVIKEILYTPY
GDIYHDTYPDFQVIIGFHGGLYDFLTKLVHLGQRDYDVVAGRWTTPNHHIWKQLN
LLPKPFNLYSFENNYPVGKIQDVAKYTTDIRSWLELFGFQLHNVLPGFPKPELEN
LELTYELLRLQTKTQEWDPGKTILGIQCELQKQLRNFISLDQLPMTPRYNDGRCL
EGGKQPRFAAVPSVFGKGIKFAIKDGIVTADIIGVANEDSRRLAAILNNAHYLEN
LHFTIEGRDTHYFIKLGSLEEDLVLIGNTGGRRILENGVNVTVSQMTSVLNG

Furin-cleavage site within the RHS-domain (red) and C-terminal domain (GHH Tox domain)in *italics*

<u>RTRRFADIQLQHGALCFNIRYGTTVE*EEKNHVLEIARQRAVAQAWTKEQRRLQEGEEGIRAWTEGEKQQLLSTGRVQGYDGYFVLSVEQYLELSDSANNIHFMR*QSEIGRR</u>

Expressed human Teneurin-1 C-terminal domain (CTD) sequence (furin cleavage site in red, GHH toxin domain in *italics*):

VTVSQMTSVLNGRTRRFADIQLQHGALCFNIRYGTTVE*EEKNHVLEIARQRAVAQAWTKEQRRLQEGEEGIRAWTEGEKQQLLSTGRVQGYDGYFVLSVEQYLELSDSANNIHFMR*QSEIGRR

Expressed human Teneurin-2 C-terminal domain (CTD) sequence (furin cleavage site in red, GHH toxin domain in *italics*):

TVSQPTLLVNGRTRRFTNIEFQYSTLLLSIRYGLTPDTLD*EEKARVLDQARQRALGTAWAKEQQKARDGREGSRLWTEGEKQQLLSTGRVQGYEGYYVLPVEQYPELADSSSNIQFLR*QNEMGKR

Purified chicken Teneurin-1 C-terminal domain (CTD) sequence (GHH toxin domain in italics):

FADIQLQHGALCFNVRYGTTVE*EEKNHVLEVARQRAVAQAWTKEQRRLQEGEEGIRAWTDGEKQQLLNTGRVQGYDGYFVLSVEQYLELSDSANNIHFMR*QSEIGRR

Purified chicken Teneurin-2 C-terminal domain (CTD) sequence (GHH toxin domain in italics):

FTNIEFQYSTLLINIRYGLTADTLD*EEKARVLDQARQRALGSAWAKEQQKARDGREGSRVWTDGEKQQLLNTGRVQGYEGYYVLPVEQYPELADSSSNIQFLR*QNEMGKR

Fig. S2. Amino acid identity and similarity between teneurin C-terminal domains and the bacterial GHH toxins. The GHH domains from three bacterial toxins were aligned with the corresponding amino acids from the C-terminal domains (CTDs) of representative teneurins (TOX) to illustrate the possibility that the teneurin CTD is an endonuclease.

HS = *Homo sapiens*
MM = *Mus musculus*
GG = *Gallus gallus*
CM = *Callorhincus milii*
Dm = *Drosophila melanogaster*

IDENTITY RESULTS

	HSTEN1TOX	HSTEN2TOX	HSTEN3TOX	HSTEN4TOX	MMTEN1TOX	MMTEN2TOX	MMTEN3TOX	MMTEN4TOX	GGTEN1TOX	GGTEN2TOX	GGTEN3TOX	GGTEN4TOX	CMTEN2TOX	CMTEN3TOX	CMTEN4TOX	CMTEN1TOX	DmTENMTOX	DmTENATOX	BrevibacillusbrevisGHHTOX	BacilluscytotoxicusGHHTOX	BacillusthuringiensisGHHTOX
HSTEN1TOX	100%																				
HSTEN2TOX	67.53%	100%																			
HSTEN3TOX	71.42%	79.22%	100%																		
HSTEN4TOX	83.11%	70.12%	75.32%	100%																	
MMTEN1TOX	93.5%	64.93%	70.12%	80.51%	100%																
MMTEN2TOX	67.53%	100%	79.22%	70.12%	64.93%	100%															
MMTEN3TOX	71.42%	79.22%	100%	75.32%	70.12%	79.22%	100%														
MMTEN4TOX	80.51%	66.23%	71.42%	94.8%	79.22%	66.23%	71.42%	100%													
GGTEN1TOX	96.1%	64.93%	68.83%	80.51%	92.2%	64.93%	68.83%	83.11%	100%												
GGTEN2TOX	64.93%	94.8%	75.32%	67.53%	64.93%	94.8%	75.32%	68.83%	67.53%	100%											
GGTEN3TOX	70.12%	79.22%	97.4%	75.32%	68.83%	79.22%	97.4%	71.42%	67.53%	76.62%	100%										
GGTEN4TOX	83.11%	71.42%	77.92%	92.2%	81.81%	71.42%	77.92%	89.61%	83.11%	71.42%	76.62%	100%									
CMTEN2TOX	63.63%	83.11%	79.22%	64.93%	62.33%	83.11%	79.22%	61.03%	61.03%	79.22%	77.92%	67.53%	100%								
CMTEN3TOX	64.93%	79.22%	90.9%	70.12%	63.63%	79.22%	90.9%	66.23%	62.33%	75.32%	90.9%	71.42%	75.32%	100%							
CMTEN4TOX	79.22%	72.72%	75.32%	87.01%	77.92%	72.72%	75.32%	84.41%	76.62%	70.12%	75.32%	89.61%	67.53%	74.02%	100%						
CMTEN1TOX	62.33%	50.64%	57.14%	59.74%	62.33%	50.64%	57.14%	58.44%	61.03%	49.35%	54.54%	59.74%	49.35%	53.24%	59.74%	100%					
DmTENMTOX	36.36%	38.96%	44.15%	38.96%	36.36%	38.96%	44.15%	37.66%	35.06%	37.66%	42.85%	38.96%	37.66%	41.55%	38.96%	38.96%	100%				
DmTENATOX	27.27%	32.46%	33.76%	33.76%	27.27%	32.46%	33.76%	33.76%	27.27%	32.46%	33.76%	32.46%	37.66%	32.46%	32.46%	27.27%	32.46%	100%			
BrevibacillusbrevisGHHTOX	23.37%	18.18%	20.77%	22.07%	24.67%	18.18%	20.77%	20.77%	22.07%	16.88%	20.77%	20.77%	18.18%	20.77%	22.07%	19.48%	16.25%	18.18%	100%		
BacilluscytotoxicusGHHTOX	14.28%	10.38%	12.98%	14.28%	16.88%	10.38%	12.98%	14.28%	14.28%	10.38%	12.98%	14.28%	10.38%	12.98%	12.98%	16.88%	8.75%	12.98%	65.43%	100%	
BacillusthuringiensisGHHTOX	33.76%	37.66%	37.66%	35.06%	36.36%	37.66%	37.66%	35.06%	33.76%	37.66%	37.66%	33.76%	33.76%	36.36%	33.76%	29.87%	35.06%	31.16%	19.48%	16.88%	100%

SIMILARITY RESULTS

	HSTEN1TOX	HSTEN2TOX	HSTEN3TOX	HSTEN4TOX	MMTEN1TOX	MMTEN2TOX	MMTEN3TOX	MMTEN4TOX	GGTEN1TOX	GGTEN2TOX	GGTEN3TOX	GGTEN4TOX	CMTEN2TOX	CMTEN3TOX	CMTEN4TOX	CMTEN1TOX	DmTENMTOX	DmTENATOX	BrevibacillusbrevisGHHTOX	BacilluscytotoxicusGHHTOX	BacillusthuringiensisGHHTOX
HSTEN1TOX	100%																				
HSTEN2TOX	76.62%	100%																			
HSTEN3TOX	81.81%	87.01%	100%																		
HSTEN4TOX	92.2%	83.11%	87.01%	100%																	
MMTEN1TOX	93.5%	76.62%	80.51%	85.71%	100%																
MMTEN2TOX	76.62%	100%	87.01%	83.11%	76.62%	100%															
MMTEN3TOX	81.81%	87.01%	100%	87.01%	80.51%	87.01%	100%														
MMTEN4TOX	89.61%	79.22%	83.11%	96.1%	84.41%	79.22%	83.11%	100%													
GGTEN1TOX	98.7%	75.32%	80.51%	90.9%	93.5%	75.32%	80.51%	90.9%	100%												
GGTEN2TOX	75.32%	98.7%	85.71%	81.81%	76.62%	98.7%	85.71%	80.51%	76.62%	100%											
GGTEN3TOX	80.51%	88.31%	97.4%	87.01%	79.22%	88.31%	97.4%	83.11%	79.22%	87.01%	100%										
GGTEN4TOX	90.9%	81.81%	87.01%	96.1%	87.01%	81.81%	87.01%	94.8%	92.2%	83.11%	85.71%	100%									
CMTEN2TOX	76.62%	90.9%	85.71%	79.22%	75.32%	90.9%	85.71%	75.32%	75.32%	89.61%	84.41%	79.22%	100%								
CMTEN3TOX	77.92%	88.31%	92.2%	84.41%	75.32%	88.31%	92.2%	80.51%	76.62%	87.01%	92.2%	83.11%	84.41%	100%							
CMTEN4TOX	87.01%	80.51%	83.11%	93.5%	81.81%	80.51%	83.11%	92.2%	85.71%	79.22%	84.41%	93.5%	76.62%	83.11%	100%						
CMTEN1TOX	71.42%	66.23%	70.12%	70.12%	68.83%	66.23%	70.12%	68.83%	71.42%	66.23%	67.53%	70.12%	62.33%	66.23%	67.53%	100%					
DmTENMTOX	49.35%	50.64%	54.54%	51.94%	49.35%	50.64%	54.54%	53.24%	50.64%	51.94%	53.24%	53.24%	49.35%	51.94%	51.94%	51.94%	103.89%				
DmTENATOX	45.45%	46.75%	50.64%	48.05%	44.15%	46.75%	50.64%	48.05%	45.45%	46.75%	49.35%	46.75%	49.35%	48.05%	46.75%	44.15%	51.94%	100%			
BrevibacillusbrevisGHHTOX	28.57%	25.97%	27.27%	27.27%	29.87%	25.97%	27.27%	25.97%	27.27%	24.67%	27.27%	24.67%	24.67%	28.57%	27.27%	27.27%	24.67%	31.16%	105.19%		
BacilluscytotoxicusGHHTOX	20.77%	20.77%	23.37%	22.07%	23.37%	20.77%	23.37%	22.07%	20.77%	20.77%	23.37%	20.77%	19.48%	24.67%	20.77%	25.97%	20.77%	22.07%	74.02%	105.19%	
BacillusthuringiensisGHHTOX	42.85%	48.05%	49.35%	46.75%	45.45%	48.05%	49.35%	48.05%	44.15%	49.35%	48.05%	46.75%	44.15%	46.75%	45.45%	49.35%	49.35%	48.05%	25.97%	24.67%	100%

Fig. S3. Purification of recombinant proteins. Lanes from Coomassie blue-stained polyacrylamide gels showing the purified CTDs of chicken teneurin-1 and teneurin-2 used for cell culture and nuclease activity studies. The size ladder indicates apparent molecular weights in kDa.

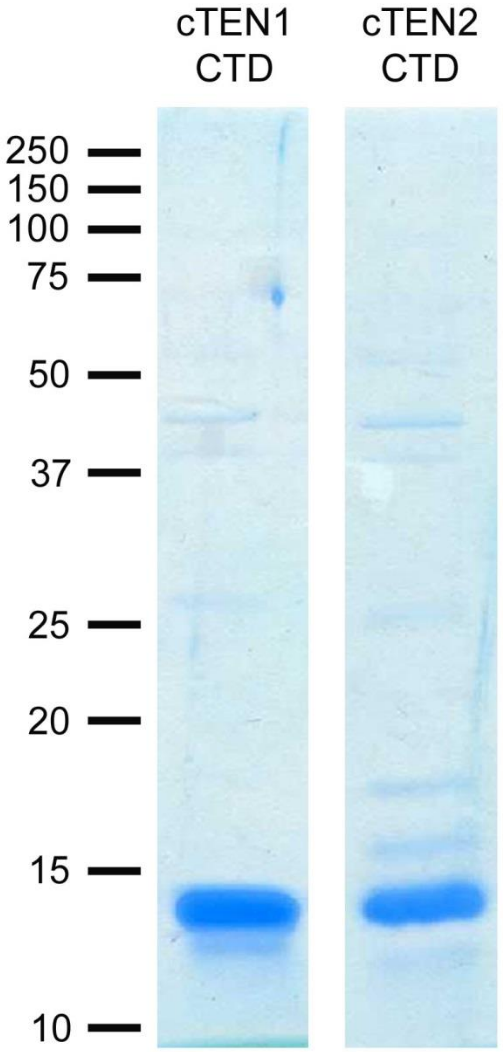

Table S1. Transcriptional profiling of HEK293 cells expressing the teneurin-2 CTD.

Affyprobes	logFC	**FC**	AveExpr	t	P.Value	adj.P.Val	geneID	Gene name
7903592	-0.72145	**0.606487**	7.354426	-12.5757	5.03E-08	8.33E-05	NM_020775	KIAA1324
7906878	-0.67236	**0.627478**	7.214012	-9.72767	7.41E-07	0.000403	NM_001014796	DDR2
7909610	-0.6658	**0.630338**	7.822956	-10.525	3.29E-07	0.000272	NM_001040619	ATF3
7914923	-0.67225	**0.627527**	8.664322	-8.30028	3.67E-06	0.000768	NM_145047	OSCP1
7918504	-0.76381	**0.588938**	8.79511	-8.34036	3.50E-06	0.000768	NM_002557	OVPG1
7919606	-0.69246	**0.618796**	9.119708	-5.54779	0.000153	0.004596	NM_001024599	HIST2H2BF
7921916	-0.59982	**0.659835**	7.830591	-9.63692	8.16E-07	0.000406	NM_003617	RGS5
7927305	1.503777	**2.835841**	9.252199	3.594072	0.003997	0.027026	NM_001144000	AGAP5
7934979	-1.16012	**0.447475**	8.087316	-15.8307	4.18E-09	1.38E-05	NM_014391	ANKRD1
7937257	-0.71473	**0.609319**	9.36582	-3.5064	0.004675	0.029707	NM_145651	SCGB1C1
7937696	-1.17705	**0.442256**	8.674062	-19.0097	5.59E-10	2.78E-06	NM_001004325	KRTAP5-2
7938263	-0.87302	**0.546001**	8.173645	-6.09672	6.72E-05	0.002971	NM_003754	EIF3F
7948332	-0.5969	**0.661171**	8.843283	-7.0881	1.69E-05	0.001556	NM_004811	LPXN
7949124	-0.71169	**0.610605**	8.297349	-7.01971	1.85E-05	0.001598	NM_005609	PYGM
7959102	-0.7667	**0.587759**	6.783417	-8.20601	4.10E-06	0.000801	NM_014365	HSPB8
7985450	-0.65561	**0.634809**	8.042572	-3.68078	0.003425	0.024381	NM_198181	GOLGA6L9
7986250	-0.64639	**0.638877**	10.61057	-9.15753	1.37E-06	0.000593	NM_001271	CHD2
7990391	-0.93391	**0.523439**	7.239254	-12.1247	7.42E-08	9.41E-05	NM_000499	CYP1A1
8012951	-0.97986	**0.50703**	8.099792	-8.85831	1.92E-06	0.000709	NM_006382	CDRT1
8018902	-1.63346	**0.322315**	8.372967	-26.4478	1.41E-11	1.40E-07	NM_173628	DNAH17
8034512	-0.66339	**0.631394**	12.72709	-3.14155	0.009025	0.043986	NR_002751	SNORD41
8044450	-0.64022	**0.641617**	8.976154	-9.88059	6.31E-07	0.000376	NM_198581	ZC3H6
8044669	-0.74997	**0.594617**	6.139221	-3.92222	0.002236	0.018966	NM_013412	RABL2A
8073015	0.590598	**1.505871**	8.841807	8.056205	4.92E-06	0.000858	NM_006855	KDELR3
8076612	-0.76622	**0.587957**	7.321342	-10.2007	4.55E-07	0.000337	NM_022785	EFCAB6
8087145	-0.67592	**0.625934**	7.738101	-8.44067	3.11E-06	0.000768	NM_001407	CELSR3
8090509	0.621983	**1.538989**	8.029379	3.589539	0.004029	0.027123	NM_004637	RAB7A
8110032	-0.59103	**0.663869**	8.910183	-7.19709	1.46E-05	0.001474	NM_153607	CREBRF
8113103	-0.77638	**0.583831**	7.527023	-10.8112	2.48E-07	0.000247	NM_001145678	KIAA0825
8113483	-0.92081	**0.528211**	8.496886	-10.5983	3.06E-07	0.000272	NM_001039763	TMEM232
8116532	-0.58961	**0.664521**	9.988008	-4.7771	0.000522	0.008636	NR_002591	SNORD95
8124448	-0.80405	**0.572738**	11.50295	-12.6511	4.72E-08	8.33E-05	NM_003543	HIST1H4H

Biology Open • Supplementary information

Table S1. Transcriptional profiling of HEK293 cells expressing the teneurin-2 CTD. (cont.)

Affyprobes	logFC	**FC**	AveExpr	t	P.Value	adj.P.Val	geneID	Gene name
8128001	-0.81901	**0.566831**	6.542142	-14.1864	1.38E-08	3.42E-05	NM_000735	CGA
8133314	-0.78137	**0.581813**	6.560076	-4.64017	0.000655	0.009589	NM_000265	NCF1
8140211	-0.66516	**0.630617**	9.648513	-4.05341	0.001778	0.016774	NR_003664	SPDYE8P
8156160	-1.61451	**0.326575**	7.891629	-7.61267	8.53E-06	0.001162	NM_017576	KIF27
8159259	-0.65752	**0.633968**	8.34969	-8.54771	2.74E-06	0.000757	NM_014582	OBP2A
8163505	0.634357	**1.552246**	9.225563	7.482806	1.01E-05	0.001236	NM_031219	HDHD3
8164766	-0.72428	**0.605298**	8.047926	-12.1016	7.57E-08	9.41E-05	NM_152572	AK8
8165496	0.649239	**1.568341**	10.8664	4.995579	0.000366	0.006937	NM_006088	TUBB4B
8165672	-1.20723	**0.433099**	6.809187	-3.32621	0.006464	0.036244	L23320	RFC

Biology Open • Supplementary information

Table S2. FASTA files used for sequence analyses.

>HSTEN1TOX
EKNHVLEIARQRAVAQAWTKEQRRLQEGEEGIRAWTEGEKQQLLSTGRVQGYDGYFVLSVEQYL
ELSDSANNIHFMR

>HSTEN2TOX
EKARVLDQARQRALGTAWAKEQQKARDGREGSRLWTEGEKQQLLSTGRVQGYEGYYVLPVEQYP
ELADSSSNIQFLR

>HSTEN3TOX
EKARILEQARQRALARAWAREQQRVRDGEEGARLWTEGEKRQLLSAGKVQGYDGYYVLSVEQYP
ELADSANNIQFLR

>HSTEN4TOX
EKARVLELARQRAVRQAWAREQQRLREGEEGLRAWTEGEKQQVLSTGRVQGYDGFFVISVEQYP
ELSDSANNIHFMR

>MMTEN1TOX
EKNHVLEMARQRAVAQAWTQEQRRLQEGEEGTRVWTEGEKQQLLGTGRVQGYDGYFVLSVEQYL
ELSDSANNIHFMR

>MMTEN2TOX
EKARVLDQARQRALGTAWAKEQQKARDGREGSRLWTEGEKQQLLSTGRVQGYEGYYVLPVEQYP
ELADSSSNIQFLR

>MMTEN3TOX
EKARILEQARQRALARAWAREQQRVRDGEEGARLWTEGEKRQLLSAGKVQGYDGYYVLSVEQYP
ELADSANNIQFLR

>MMTEN4TOX
EKVRVLELARQRAVRQAWAREQQRLREGEEGLRAWTDGEKQQVLNTGRVQGYDGFFVTSVEQYP
ELSDSANNIHFMR

>GGTEN1TOX
EKNHVLEVARQRAVAQAWTKEQRRLQEGEEGIRAWTDGEKQQLLNTGRVQGYDGYFVLSVEQYL
ELSDSANNIHFMR

>GGTEN2TOX
EKARVLDQARQRALGSAWAKEQQKARDGREGSRVWTDGEKQQLLNTGRVQGYEGYYVLPVEQYP
ELADSSSNIQFLR

>GGTEN3TOX
EKARILEQARQRALSSAWAREQQRVRDGEEGARLWTEGEKRQLLSAGKVQGYDGYYVLSVEQYP
ELADSANNIQFLR

>GGTEN4TOX

Biology Open • Supplementary information

EKARVLELARQRAVAQAWAREQQRLRDGEEGIRSWTEGEKQQVLNTGRVQGYDGYFVISVEQYP
ELSDSANNIHFMR

>CMTEN2TOX
EQARLLEQARLRALANAWTREQQKARDGKEGGRLWTEGEKQQLLSAGRIQGYEGYYVLPVEQYP
ELADSSTNIQFLR

>CMTEN3TOX
EKARILEQARQRALGCAWAREQQRVRDGEDGVRLWTEGEKRQLLSAAKVQGYDGYYVLSVEQYP
ELADSVSNIQFLR

>CMTEN4TOX
EKARVLELARQRAVTQAWAREDQRLRDGEEGVRSWTEGEKQQLLSTGRVQGYDGYFVTTVEQYP
ELSDSVNNIHFMR

>CMTEN1TOX
ERRHVLELAGQRAVARAWARERRRLGRGERGTRAWTEAERRQLLAGGRVPGYDGRAVLPPEQFA
ELSDSPGNVRFVR

>DmTENMTOX
ERHRILKHAHKRAVERAWELEKQLVAAGFQGRGDWTEEEKEELVQHGDVDGWNGIDIHSIHKYP
QLADDPGNVAFQRDAK

>DmTENATOX
EQQRLMHHAKLTAVRKAWHREKEALRSGLTTALEWSQQETDEILKQSYANNYEGEYIHDVNLYP
ELAEDPYNIKFVK

>BrevibacillusbrevisGHHTOX
EWNRRLAKRRDAGVKEFWKQEKRRIKNGEPTTRNWTQEQKDAILSNQVPTFNGKPITGHHAYSV
SKYPHLANRGEIIYPVT

>BacilluscytotoxicusGHHTOX
EWNKKFAKRWDKGVKEFWYQEQQRILNNEPLTREWSQEQLKDILAGKTPKFDGKPIAGHHSYSA
AKYPHLADKGEIIYPVT

>BacillusthuringiensisGHHTOX
LTDKQFSALRQKAVRHAWRQEKELIKKTGKGTRDWTPAEKLELLQTGKVKGYEGQHMKSANEYP
DFAGEPDNIQFLK

Biology Open • Supplementary information
